# Functional divergence and adaptive selection of *KNOX* gene family in plants

**DOI:** 10.1515/biol-2020-0036

**Published:** 2020-06-14

**Authors:** Lingyan Meng, Xiaomei Liu, Congfen He, Biyao Xu, Yaxuan Li, Yingkao Hu

**Affiliations:** College of Life Sciences, Capital Normal University, Beijing, 100048, China; Beijing Key Lab of Plant Resource Research and Development, Beijing Technology and Business University, Beijing, 100048, China

**Keywords:** functional divergence, *KNOX*, phylogenetic tree, positive selection, segmental duplication

## Abstract

KNOTTED-like homeodomain (*KNOX*) genes are transcriptional regulators that play an important role in morphogenesis. In the present study, a comparative analysis was performed to investigate the molecular evolution of the characteristics of the *KNOX* gene family in 10 different plant species. We identified 129 *KNOX* gene family members, which were categorized into two subfamilies based on multiple sequence alignment and phylogenetic tree reconstruction. Several segmental duplication pairs were found, indicating that different species share a common expansion model. Functional divergence analysis identified the 15 and 52 amino acid sites with significant changes in evolutionary rates and amino acid physicochemical properties as functional divergence sites. Additional selection analysis showed that 14 amino acid sites underwent positive selection during evolution, and two groups of co-evolutionary amino acid sites were identified by Coevolution Analysis using Protein Sequences software. These sites could play critical roles in the molecular evolution of the *KNOX* gene family in these species. In addition, the expression profiles of *KNOX* duplicated genes demonstrated functional divergence. Taken together, these results provide novel insights into the structural and functional evolution of the *KNOX* gene family.

## 1 Introduction

Homeotic genes are the main genes that regulate the development of organisms. They represent a class of transcription factors (TFs) containing a highly conserved homeobox of 183 bp, which encodes a typical DNA-binding domain of 60 amino acids, also known as a homeodomain (HD). The first cloned homeobox gene was from *Drosophila* [1]. The highly homologous sequence of Knotted-1 (Kn1) to animal homeoboxes was detected in maize by transposon tagging [2]. Homeobox genes are widely found in eukaryotes [3]. Genes encoding homologous proteins are classified into two classes: three amino acid length extension (TALE) and non-TALE [4]. Four types of *TALE* genes have been identified in animals: *MEIS*, *IRO* (*Iroquois*), *TGIF*, and *PBC*. Furthermore, according to differences in characteristic domains and functions, there are two types in plants: *KNOX* (*KNOTTED-like homeobox*) and *BELL* (*BEL-Like*) [5].

*KNOX* proteins can form heterodimers with BELL in the TALE superclass [3]. *KNOX* includes four domains: a C-terminal homeodomain (HD), *KNOX1* and *KNOX2* at the conserved N-terminal region, and an ELK domain upstream of the homologous domain. Owing to the similarity between the MEIS and *KNOX* family structures, the *KNOX1* and *KNOX2* domains are also known as the MEINOX domain, and there are three additional amino acids (P–Y–P) between the first and second helices in their homeobox [5,6]. In addition, the ELK domain, which can function as a nuclear localization signal (NLS), spans ∼21 amino acids rich in glutamic acid (Glu, E), leucine (Leu, L), and lysine (Lys, K) [7]. Between the ELK and *KNOX2* domains is the GSE domain, which is rich in proline (Pro, P), glutamic acid (Glu, E), serine (Ser, S), and threonine (Thr, T). The residue sequence (PEST sequence) regulates protein stability and degrades its encoded protein through the ubiquitin degradation pathway [8]. Furthermore, Kerstetter et al. [9] classified the *KNOX* gene family into class I and class II *KNOX* subfamilies based on structural features, phylogenetic relationships, and expression patterns.

*KNOX* genes have been isolated from many plants, such as *Nicotiana tabacum* [10], *Arabidopsis thaliana* [11,12], *Solanum lycopersicum* [13], *Medicago truncatula* [14], and *Physcomitrella patens* [15]. In most monocots, the *KNOX1* gene is expressed only in shoot apical meristems (SAM) and not in the primordium. In compound-leaf species, *KNOX1* are expressed in both SAM and the leaf primordium [16], showing that they may play a significant role in maintaining diversity in leaf morphology [3]. The *KNOX2* gene regulates the morphological transformation of haploid to diploid cells in terrestrial plants [17].

In *A. thaliana*, the class I subfamily includes *STM* (SHOOT MERISTEMLESS), *KNAT1*, *KNAT2*, and *KNAT6* [18], and the class II subfamily includes *KNAT3*, *KNAT4*, *KNAT5*, and *KNAT7* [3,19], which are widely distributed. *STM* and *KNAT1* are used to establish and maintain SAM. Similarly, *KNAT6* has previously been shown to function in the maintenance of borders during SAM and embryogenesis [20]. Furthermore, *KNAT1* promotes inflorescence development, while *KNAT2* regulates flower type [8,18,21]. The class I gene *STM* regulates the development of the plant meristem in *Arabidopsis* [8], and regulation of gene expression leads to the petal spurs rapidly evolving in *Antirrhinum* [22]. In summary, the class I *KNOX* gene is involved in the morphogenesis of lateral organs and maintains the function of SAM and the diversity of leaf morphology [3,22].

Meanwhile, the *KNOX* class homeobox genes *Oskn2* and *Oskn3* in rice are both expressed in the tissues of the SAM and participate in the regulation of SAM formation. For instance, class II *KNOX* genes, such as *KNAT3*, *KNAT4*, and *KNAT5,* contribute to the differentiation of tissues in organs in *Arabidopsis* [9,23,24]. The regulatory network within which *KNAT7* functions contributes to the negative regulation of *Arabidopsis* and *Populus* secondary cell wall biosynthesis [24,25]. The class II subfamily lacks phenotypic due to mutations; however, there have been relatively few previous studies. In brief, *KNOX* genes are involved in the growth and development of different tissues and organs in different species [26,27,28]. Plants must constantly adjust their physiological processes to adapt to changes in the external environment [29]. TFs are considered to be key targets for studying the molecular mechanisms of abiotic stress response because they, either alone or collectively, regulate the expression of many downstream target genes [30].

In the present study, we identified *KNOX* genes in different species and classified them by reconstructing phylogenetic trees. Then, we identified the critical amino acid sites responsible for functional divergence, positive selection, and co-evolution. Together with expression profiles, we present some insights into the molecular evolution of the *KNOX* gene family, which can be useful for future research on the functions of these genes.

## 2 Materials and methods

### 2.1 Identification of plant *KNOX* gene family

Genes from the plant *KNOX* gene family were identified from 10 species that represented monocotyledonous, dicotyledonous, and bryophyte plants. The *KNOX* gene family members from the *Arabidopsis* genome were obtained from the TAIR database (http://www.arabidopsis.org/) and then BLAST searched as seed sequences in the Phytozome database (http://www.phytozome.org) to obtain homologous sequences from nine other species (*Glycine max*, *Populus trichocarpa*, *Gossypium raimondii*, *Solanum lycopersicum*, *Oryza sativa*, *Brachypodium distachyon*, *Sorghum bicolor*, *Zea mays*, and *Physcomitrella patens*). If the E value of the sequence was ≤1 × 10^−5^, then it was listed as a candidate sequence. The Pfam (http://pfam.xfam.org) and SMART (http://smart.embl-heidelberg.de/) online tools were used to determine whether the candidate sequence contained the *KNOX1*, *KNOX2*, ELK, and HD to ensure that the sequence domain was intact and used for the next analysis. In addition, coding sequences, protein sequences, and genomic sequences of *KNOX* family members were downloaded from the Phytozome database. The physicochemical properties of the *KNOX* gene family were obtained from the ExPASy database (https://www.expasy.org/), including the amino acid number, isoelectric point (PI), and molecular weight (MW) of the protein [31].

### 2.2 Phylogenetic tree construction

Multiple sequence alignment of the sequences from *KNOX* family members was performed with the MUSCLE program [32,33]. Three methods were used to construct the phylogenetic tree: Bayesian phylogenetic trees in MrBayes 3.2.5 [34] and neighbor-joining (NJ) and maximum-likelihood (ML) trees in MEGA 7.0 [35]. The reliability of interior branches was assessed with 1,000 bootstrap samples [35].

### 2.3 Exon–intron structure and motif analysis

The exon–intron structure was analyzed using the online tool GSDS (http://gsds.cbi.pku.edu.cn/) with the coding sequences (CDS) and genomic sequences of *KNOX* family members [36]. Conserved motifs of *KNOX* family members were identified using the online tool MEME (http://meme-suite.org/tools/meme) [37]. The maximum number of motifs = 10, and the remaining parameters were set to the default settings.

### 2.4 Duplication event analysis

Tandem duplication and segmental duplication were used to determine the main amplification methods of the *KNOX* gene family. The synonymous substitution rates (*K*_s_) of gene pairs produced by segmental repeat events were identified using the Plant Genome Duplication Database (http://chibba.agtec.uga.edu/duplication) [38]. To avoid the risk of saturation and improve the accuracy of the results, the *K*_s_ value greater than 1 and anchors less than 3, the approximate age of the segmental duplication event was estimated by the following formula: *T* = *K*_s_/2*λ* [39]. The synonymous substitutions per year (*λ*) were 1.5 × 10^−8^ for *Arabidopsis* [40], 6.1 × 10^−9^ for *Glycine max*, 6.5 × 10^−9^ for *Brachypodium distachyon* [41], 9.1 × 10^−7^ for *Populus trichocarpa* [42], 1.5 × 10^−8^ for *Gossypium raimondii* [43], 6.5 × 10^−9^ for *Oryza sativa*, 6.1 × 10^−9^–6.5 × 10^−9^ for *Sorghum bicolor* [44], and 6.5 × 10^−9^ for *Zea mays* [45].

### 2.5 Functional divergence analysis

DIVERGE 3.0 was used to detect the functional divergence between clusters of the *KNOX* gene family [46]. The extent of divergence can be measured using the type I (site-specific altered selective constraints) and type II (radical shift in amino acid physiochemical properties) functional divergence coefficients (θI and θII) between subfamilies [47,48,49]. Moreover, Bayesian posterior probability (*Q*_k_) can detect specific amino acid sites where functional divergence has occurred. In our study, the threshold of *Q*_k_ was set to 0.9.

### 2.6 Positive selection analysis

Positive selection was investigated using the maximum likelihood approach in the CODEML procedure in PAML [50,51]. Site models, including null models (M0 and M3) and alternative hypothesis models (M7 and M8), were implemented in this program. A detailed description of the positive selection site test method can be found in Wang et al. [52].

### 2.7 Coevolution analysis

Coevolution analysis using protein sequences (CAPS) was performed with PERL-based software [53]. A detailed description of the coevolution sites test method can be found in Song et al. [54].

### 2.8 Protein structure prediction

The 3D structure of the *KNOX* protein was predicted using online software PHYRE2 (http://www.sbg.bio.ic.ac.uk/phyre2/html/page.cgi?id=index) [55]. We used protein sequences to construct the 3D structure of *KNOX* family member AT4G08150 and then to screen important amino acids sites that were labeled on the 3D structure.

### 2.9 Expression analysis of *KNOX* genes

RNA-Seq data were introduced to further analyze the expression of plant *KNOX* genes. The *Arabidopsis* eFP Browser (http://bar.utoronto.ca/efp/cgi-bin/efpWeb.cgi) tool and the rice eFP Browser (http://www.bar.utoronto.ca/efprice/cgi-bin/efpWeb.cgi) tool were used to search data from *Arabidopsis* and rice, respectively. A heat map was generated using the TBtools program [56].

## 3 Results

### 3.1 Identification of *KNOX* gene family

Nine *KNOX* genes of *Arabidopsis* were obtained from the TAIR database. The gene *AT1G14760* was not analyzed because it contains only two *KNOX* domains, which belong to the KNATM class, that were not included in this analysis. Furthermore, 121 candidate *KNOX* gene sequences from nine other species were obtained through BLAST searches in the Phytozome database (Table A1). The Pfam [57] and SMART [58] online tools were used to ensure the wholeness of the domains (*KNOX1*, *KNOX2*, ELK, and HD). The results showed that the domains were complete, and a total of 129 typical *KNOX* family members were identified. The relevant information groups, gene IDs, protein lengths, isoelectric point of the deduced polypeptides, and molecular weight of the *KNOX* genes are listed in Table A2. The average number of amino acid residues ranged from 215 to 636 (average 345), and the isoelectric point of the *KNOX* gene family ranged from 5.12 to 9.53 (average 6.62). Except for GRMZM2G433591, all other members were weakly acidic. The average molecular mass of the *KNOX* family ranged from 28,353.8 to 72,405.5 Da (average 38,715.4 Da).

### 3.2 Phylogenetic analysis of the *KNOX* gene family

**Figure 1:**
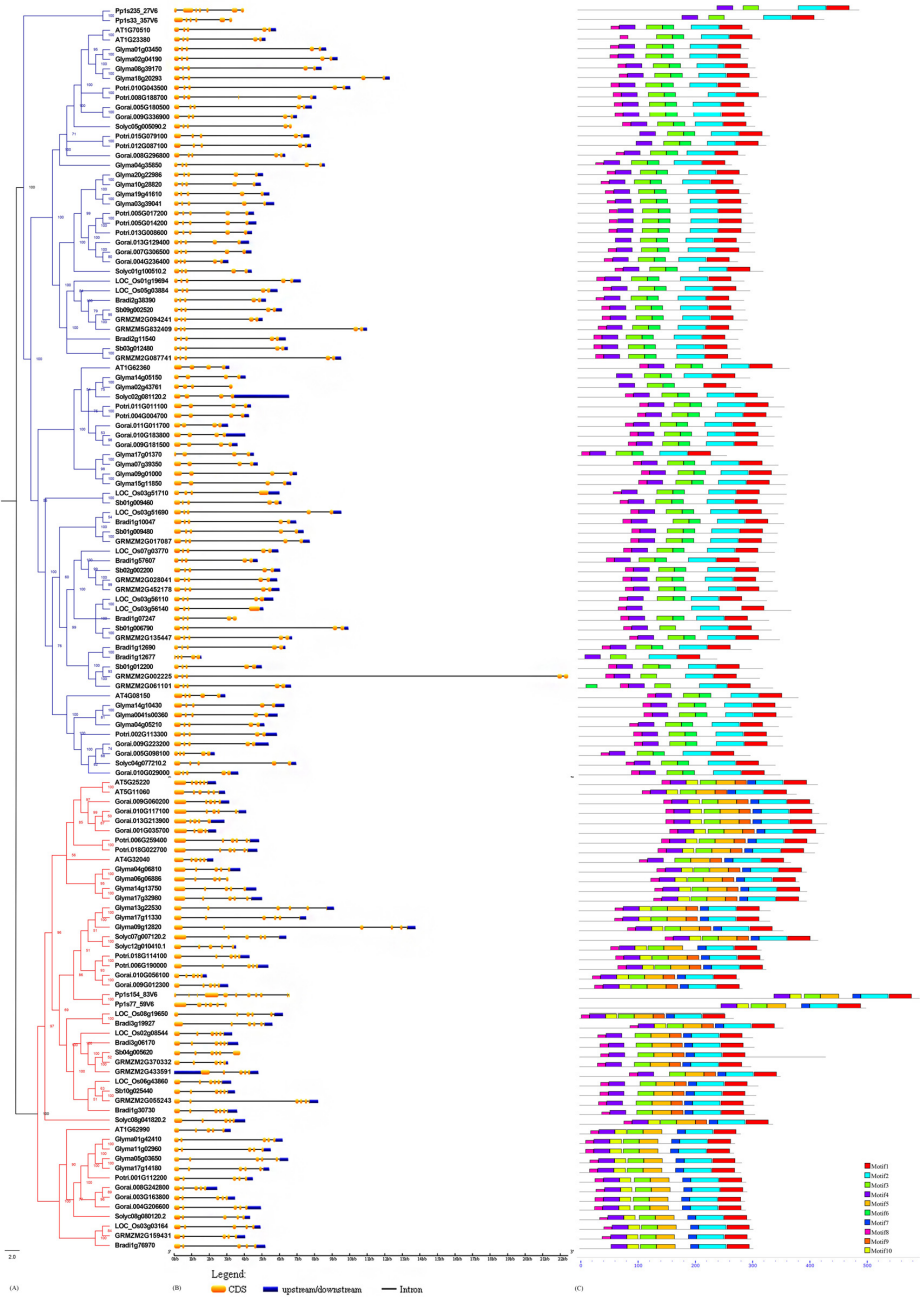
Phylogenetic relationship, exon–intron structure, and motif structure of plant *KNOX* gene family members. (a) The rooted Bayesian phylogenetic tree. The branches of the two different colors represent different subfamilies: blue represents the class I *KNOX* gene and red represents the class II *KNOX* gene. (b) Exon and intron structure of *KNOX* genes. Yellow boxes, exons; lines, introns. The lengths of boxes and lines are scaled according to gene length. (c) MEME motif structures. Numbers and different colors were used to represent conservative motifs.

**Figure A1:**
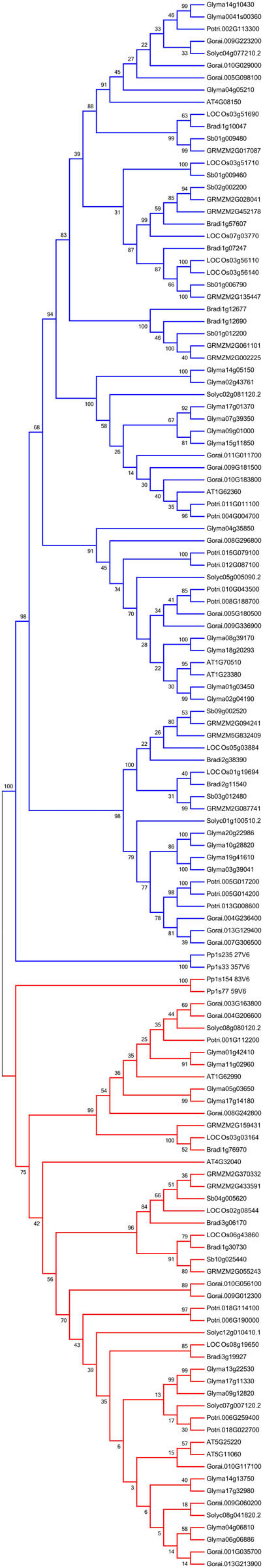
Neighbor-joining phylogenetic tree of the KNOX gene family. Two branches of different colors represent different subfamilies: blue represents class I KNOX genes, and red represents class II KNOX genes.

To investigate the phylogenetic relationships of *KNOX* genes in 10 plant species (*Arabidopsis thaliana*, *Glycine max*, *Populus trichocarpa*, *Gossypium raimondii*, *Solanum lycopersicum*, *Oryza sativa*, *Brachypodium distachyon*, *Sorghum bicolor*, *Zea mays and Physcomitrella patens*), we used the MUSCLE software [32,33] to perform multiple sequence alignments of 129 protein sequences and then used three methods to construct phylogenetic trees: neighbor-joining (Figure A1), maximum-likelihood (data not displayed), and Bayesian inference [34,35] (Figure 1a). According to the results, the topology of the three methods was consistent; the subsequent study uses the Bayesian phylogenetic tree. The 129 homeobox genes from 10 species, including monocots and dicots and *P. patens*, were divided into two subfamilies: class I and class II [59]. The sequence analysis and expression pattern analysis supported this result, which was also consistent with other previous research [9]. The class I subfamily includes 80 members, while the class II subfamily includes 49 members. This difference may be due to the method of gene amplification, which leads to the difference in the number of subfamily members.

Figure 1b shows the exon–intron structure of *KNOX* gene family members analyzed by the GSDS online system [60]. Most members of the subfamily contained five exons. The number of exons was conservative within the subfamily, and there was no significant difference in the number of exons in the same subfamily. Of the class I members, 78.75% contained five exons, whereas 13 members contained four exons (Figure 1b), and 91.84% of the members of the class II subfamily contained five exons. The number of exons in *P. patens* was significantly different. For example, Ppls154_83V6 contained 10 exons, indicating that exons may have been lost during evolution to adapt to the environment.

The conserved domains of the *KNOX* gene family were analyzed using the MEME online tool [37], and 10 conserved motifs were obtained that were named motif1–motif10. The structure and order of these motifs in the two subfamilies are shown in Figure 1c. Motifs 8, 4, 3, 2, and 1 were widely distributed among species in that order, except for *P. patens*, which did not contain motif 8. However, there were also slight differences between different members of the same plant. Most members of the class I subfamily contained motifs 8, 4, 3, 6, 2, and 1 arranged in that order, and most members of the class II subfamily contained motifs 8, 4, 10, 3, 5, 9, 7, 2, and 1 arranged in that order. Differences in motifs may be an important cause of functional divergence in the two subfamilies.

### 3.3 Expansion analysis of plant *KNOX* gene family

Gene duplication is a major driving force of adaptive evolution in species [61]. In this study, we investigated the gene duplication mode of the *KNOX* gene family and mainly studied tandem duplication and segmental duplication. Tandem duplication gene pairs were detected in only three species; all of which were members of the monocotyledonous of the class I subfamily. Furthermore, segmental duplication genes were clearly detected in eight species (Table A3). Of the segmental duplication gene pairs, 93% were detected in dicotyledons, whereas only four *KNOX* segmental duplication gene pairs were detected in monocotyledons. We found that the class I subfamily contained both segmental and tandem duplication genes, which may explain the higher number of genes in class I than class II. In dicotyledonous plants, genes are mainly amplified through segmental duplication; in monocotyledonous plants, tandem duplication and segmental duplication coexist. To estimate the approximate time of segmental duplication events, the base synonymous mutation rate (*K*_s_-values) was used [38] (Table A3). The results showed that the segmental duplication events of most species were consistent with the large-scale duplication events, and segmental duplications were preserved after genome duplication.

### 3.4 Functional divergence analysis of *KNOX* gene family

To determine the difference in the evolutionary rates and physicochemical properties of amino acid sites, the type I and II functional divergence of the two subfamilies was estimated using DIVERGE [48,62]. Key amino acid sites for functional divergence were determined based on posterior probability (*Q*_k_). The results in Table 1 show that the divergence coefficients of type I of the two subfamilies were significant (θI = 0.442 ± 0.052; LRT = 71.696; *P* < 0.01), indicating that the amino acid sites between the two subfamilies have different evolutionary rates. Meanwhile, the type II coefficients of the two subfamilies were also significant (θII = 0.106 ± 0.186; *P* < 0.01), indicating the possible presence of type II divergence sites during evolution between the two subfamilies. Furthermore, the amino acid sites were analyzed between groups under stringent conditions (*Q*_k_ > 0.9) to confirm the amino acid sites where functional divergence had occurred [48].

**Table 1:** Functional divergence between subfamilies of the plant *KNOX* gene family

Group1	Group2	Type I			Type II	
		θI ± s.e.	LRT	*Q*_k_ > 0.9	θII ± s.e.	*Q*_k_ > 0.9
Class I	Class II	0.442 ± 0.052	71.696**	15	0.106 ± 0.186	52

Note: θI and θII, the coefficients of type-I and type-II functional divergence between class I and class II. LRT: likelihood ratio test. **P < 0.01, highly significant. *Q*_k_: posterior probability.

The results identified 15 sites with a high probability of being associated with type I functional divergence. There were 52 type II functional divergence sites (Table 2), more than twice the sites identified for type I, of which eight points (140A, 155Q, 170A, 223I, 224R, 283H, 286K, and 345Q) occurred in both type I and type II functional divergence, indicating that they underwent changes in evolutionary rates and physicochemical properties simultaneously. Therefore, these sites are expected to play an important role in functional differences during evolution. Apart from this, the number of type I and type II functional divergence sites was different, and more critical amino acid sites were identified as type II functional divergence within each subfamily. Hence, the functional divergence between genes of the two subfamilies was attributed primarily to rapid changes in amino acid physiochemical properties, followed by a shift in evolutionary rates.

**Table 2:** Functional divergence sites between subfamilies of the plant *KNOX* gene family

	Amino acid sites
Type I	138I, **140A**, **155Q**, 164U, **170A**, 198M, 213T, 222F, **223I**, **224R**, 229Q, **283H**, **286K**, **345Q**, 362P
Type II	133A, 137K, **140A**, 145S, 146T, 151Y, 153D, **155Q**, 158G, 159A, 161P, 163V, 166R, 169A, **170A**, 171R, 175E, 196Q, 211E, 214R, 215P, 217Q, 220M, 221E, **223I**, **224R**, 225R, 253S, 256E, 257E, 278R, 282N, **283H**, 285L, **286K**, 287K, 300S, 305K, 310K, 312A, 313R, 317L, 318T, 322L, 324Y, 332S, 336A, 340S, 344D, **345Q**, 359H, 372D

Note: amino acid sites in bold font indicate that they were responsible for both type I and type II functional divergence.

### 3.5 Positive selection and co-evolution in *KNOX* gene family

The site model was selected to determine the selection pressure on different amino acid codon sites [51]. The results are shown in Table 3. The selection pressure was significantly different between M0 (one-ratio) and M3 (discrete; *P* < 0.01). The M3 model was better than the M0 model, indicating that different sites experience different selection pressures. The 2Δ ln *L* of M7 (beta) vs. M8 (beta & *ω* > 1) was 5795.66, the likelihood ratio test result was extremely significant (df = 2, *P* < 0.01), and the M8 model had an *ω* value of 2.63459, much >1, indicating that 14 amino acid positions were strongly affected by positive selection. Table 3 shows the positive selection sites with a posterior probability >95%. Among them, 143H, 171R, and 228S were significant positive selection sites, and 130D, 133A, 134M, 140A, 149Q, 165D, 172Q, 232M, 315K, 318T, and 322L were extremely significant positive selection sites.

**Table 3:** Positive selection analysis among *KNOX* genes using site-specific models

Model	InL^a^	2Δ*l* \textcolor[rgb]{0,0,0}{2}\textcolor[rgb]{0,0,0}{\triangle }\textcolor[rgb]{0,0,0}{{l}}	Estimate of parameters	Positively selected sites^b^
M0	−25111.04	632.77**(M0 vs. M3)	*ω* = 0.07564	Not allowed
M3	−24478.27		*p*_0_ = 041377, *ω*_0_ = 0.00271, *p*_1_ = o.37838, *ω*_1_ = 0.05236, *p*_2_ = 0.20785, *ω*_2_ = 0.20938	None
M7	−24434.60	5795.66**(M7 vs. M8)	*p* = 0.63964, *q* = 6.96441	Not allowed
M8	−30230.26		*p*_0_ = 0.99999, *p* = 0.98088, *q* = 1.39062, *p*_1_ = 0.00001, *ω* = 2.63459	130D**, 133A**, 134M**, 140A**, 143H*, 149Q**, 165D**, 171R*, 172Q**, 228S*, 232M**, 315K**, 318T**, 322L**

Note: ^a^ log likelihood. ^b^ positive selection sites are inferred at posterior probabilities >95%. *P < 0.05; **P < 0.01. Amino acid sites in bold font also found to be involved in the functional divergence.

We used CAPS, which is significantly more sensitive than other methods, to analyze coevolved amino acid residues in the *KNOX* gene family [53]. We found two groups of coevolved sites: 248S and 249D, and 382L and 383Y. All sites were labeled according to their 3D structure to further investigate their interdependence (Figure 3).

### 3.6 Three-dimensional structure prediction and critical amino acid site identification of plant *KNOX* proteins

**Figure 2:**
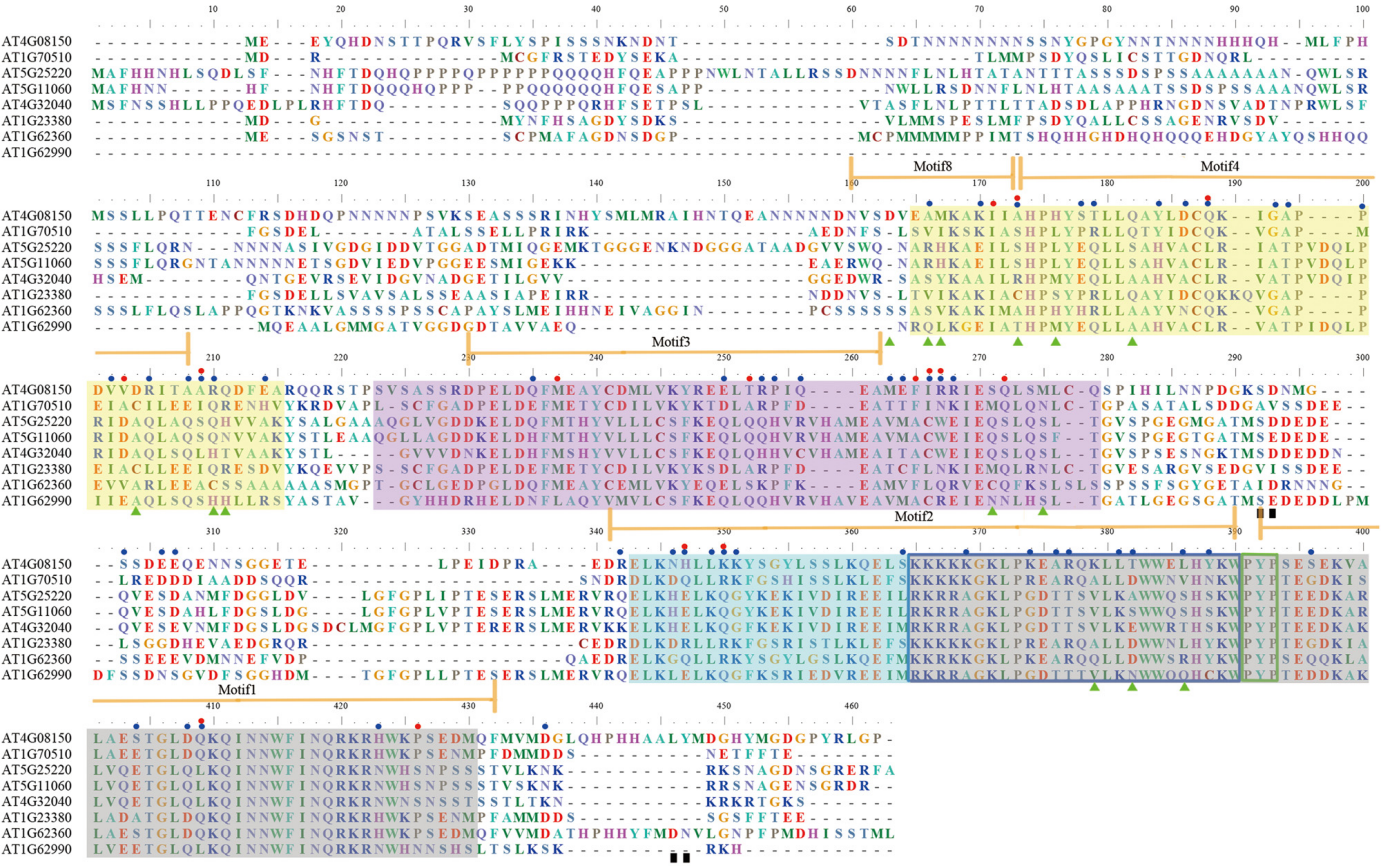
Multiple sequence alignment of *Arabidopsis KNOX* sequences. Typical domains *KNOX1*, *KNOX2*, ELK, and HD of *KNOX* protein are marked by yellow, purple, blue, and grey shadows, respectively. Motifs 1–4 and motif 8 are indicated with brown arrows above sequences. The amino acid sites of type I and II functional divergences, positive selection, and co-evolution are labeled, respectively, with blue circles, red circles, green triangles, and black boxes. Blue and green frames indicate the first α-helix and PYP loop, respectively.

**Figure 3:**
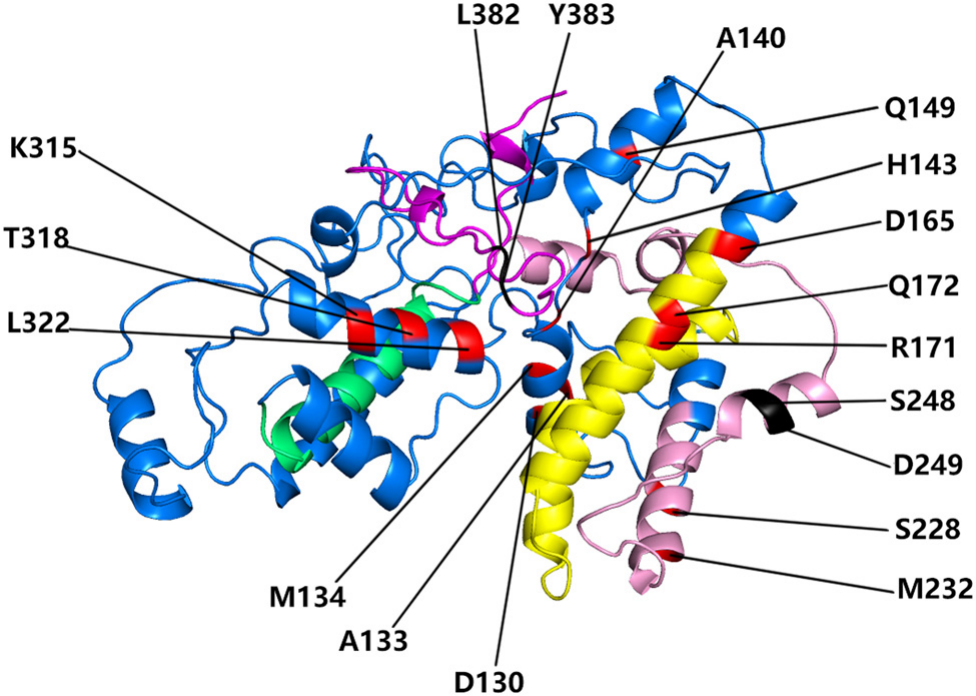
Model building of *KNOX* protein 3D structure. This figure was produced using Chimera software, and amino acids refer to the AT4G08150 sequence. The HD, ELK, *KNOX2*, and *KNOX1* domains are in yellow, pink, lime green, and magenta, respectively. The red indicates those that had undergone positive selection, and black indicates amino acid sites identified by the co-evolution analysis.

We used PHYRE2 to predict the 3D structure of the *KNOX* family member AT4G08150 [55,63]. The critical amino acid sites were displayed by the multiple sequence alignment and 3D structure (Figures 2 and 3). These 14 sites were mainly dispersed on the *KNOX1* domain, two positive selection sites were distributed on the *KNOX2* domain, and three positive selection sites were distributed on the HD first alpha helix. The results indicated that the *KNOX1* domain was more susceptible to positive selection pressure during the evolution of the *KNOX* gene family. Amino acid position 140A has undergone both functional divergence and positive selection and was located in the *KNOX1* domain and at the C-terminus of motif 8 (Figure 2). The two pairs of co-evolutionary sites we detected were marked on the 3D structure (Figure 3). We found that two sets of positive selection sites were located on the C-terminal non-functional domain and were close to each other, showing that they may play a certain role in maintaining the spatial structural stability of *KNOX* proteins.

### 3.7 Expression analysis of *KNOX* gene family

**Figure 4:**
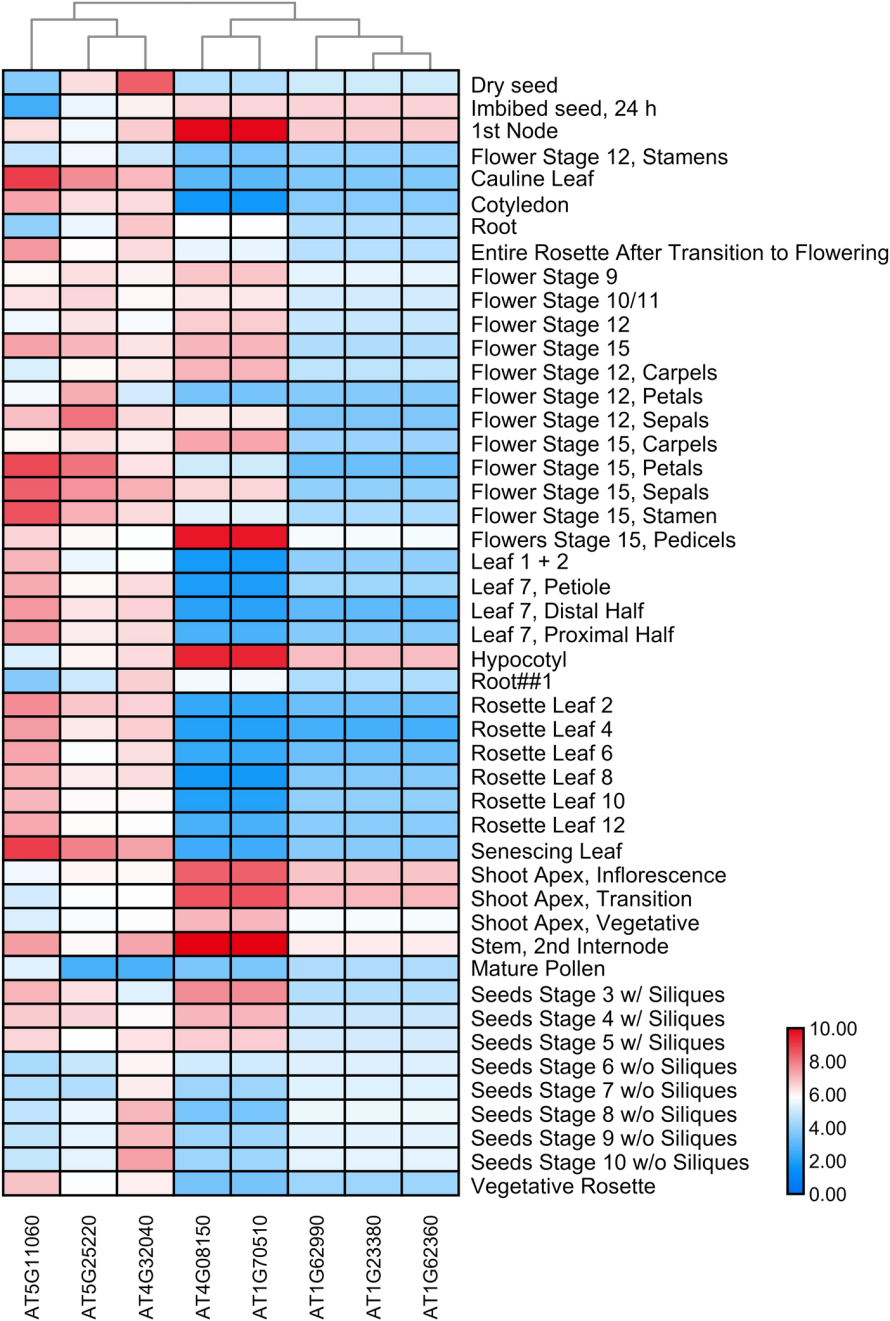
Expression profiles of *Arabidopsis thaliana KNOX* genes. The expression level is represented by a color: dark red indicates the highest expression level and dark blue indicates the lowest expression level. Other colors indicate medium levels of expression.

**Figure 5:**
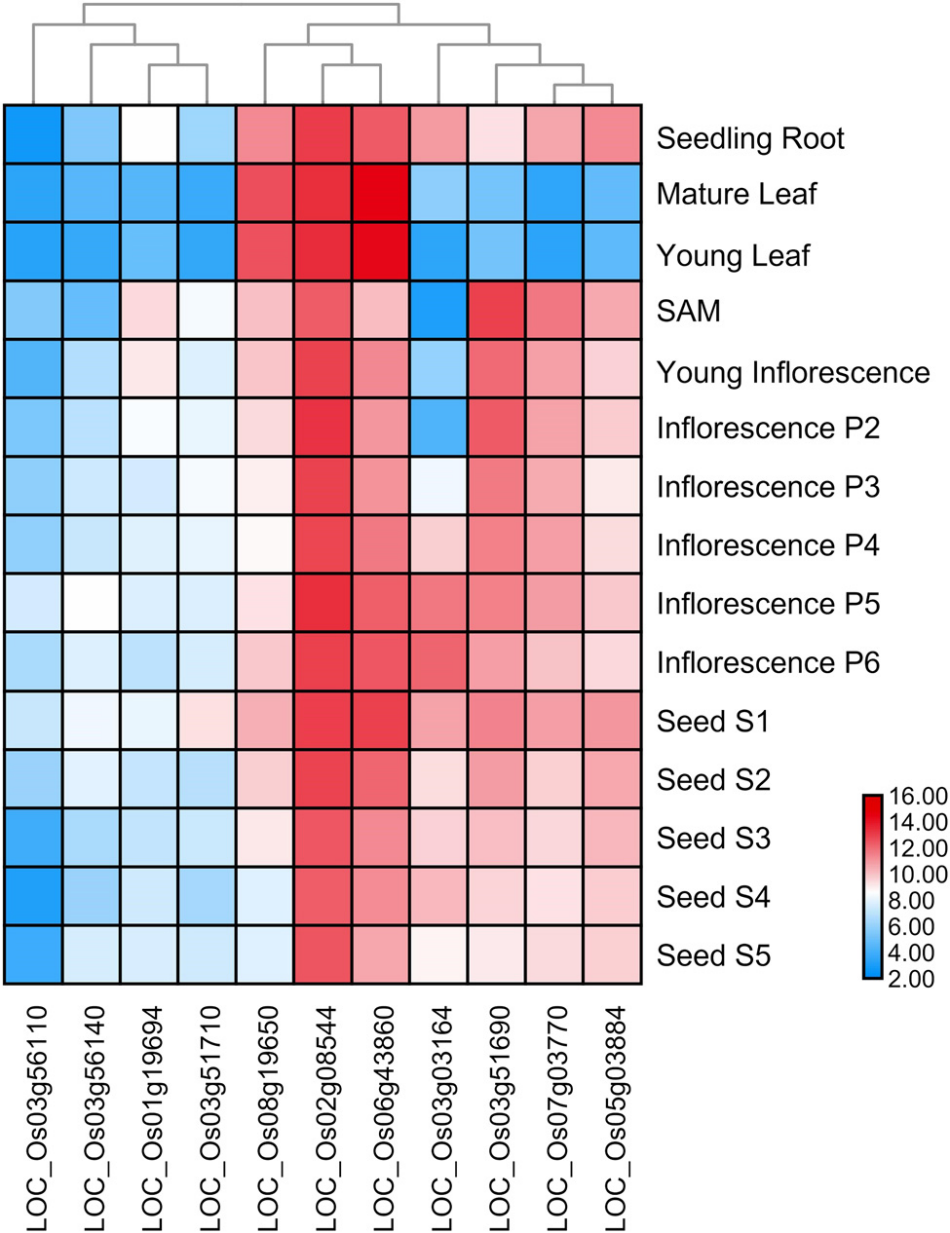
Expression profiles of rice *KNOX* genes. The expression level is represented by a color: dark red indicates the highest expression level, and dark blue indicates the lowest expression level. Other colors indicate medium levels of expression.

To investigate the expression patterns of homologous *KNOX* genes in subgroups involved in plant growth and development, a heat map was constructed using TBtools (Figures 4 and 5). Members of the same subfamily exhibited similar transcription abundance profiles; however, there were also members that had similar expression profiles but unique phylogenies, such as AT1G62990. It should be noted that AT4G08150 and AT1G70510 belong to the same subfamily (class I). They are expressed at high levels in the pedicels, hypocotyls, and stem but at lower levels in cotyledons and leaves. From the overall expression level, the higher expression levels of AT1G23380 and AT1G62360 in shoots may be related to their indispensability for the formation and maintenance of SAM [29]. These results suggested that members in the same subfamily may play similar roles in the same organization. AT5G11060, AT5G25220, and AT4G32040 are from the class II subfamily. Their overall transcription was richer than that of the class I subfamily. The expression level was higher in senescing leaves. AT4G32040 was highly expressed in dry seeds. AT5G11060 was more highly expressed in leaves and different stages of flowers (Figure 4). LOC_Os02g08544 and LOC_Os06g43860 are also from the class II subfamily, and both of them were highly expressed in all tissues and organs (Figure 5). Three members, LOC_Os02g08544, LOC_Os06g43860, and LOC_Os08g19650, were highly expressed in mature and young leaves, whereas the other members exhibited relatively low expression levels. In addition, the expression of LOC_Os03g51690, LOC_Os07g03770, and LOC_Os05g03884 was higher in the seeds but lower in the leaves, which indicates that sub-functionalization had occurred.

## 4 Discussion

### 4.1 Genomic analysis of the *KNOX* gene family

In the present study, we isolated 129 candidate *KNOX* gene sequences after removing incomplete and redundant sequences from 10 different species. There were nine from *Arabidopsis* [64] and eight from *Solanum lycopersicum* [65], which are consistent with the results of previous studies. Genome-wide analysis showed that the *KNOX* gene family was divided into two subfamilies: class I and class II (Figure 1). Both subfamilies contain monocots, dicots, and *P. patens*. Class I subfamily *KNOX* genes are similar to zmkn1 and are mainly expressed on the SAM of monocots and dicots [3,66]. According to previous studies, only one *KNOX* gene had evolved before the emergence of terrestrial plants, indicating that *KNOX* genes originated during the divergence of the last common ancestor of moss and vascular plants. *KNOX* genes are divided into four domains: *KNOX1*, *KNOX2*, ELK, and HD [64,67]. The *KNOX1* domain has negative regulatory effects on the transcription of target genes. The *KNOX2* domain mediates the interaction between *KNOX* and members of the *BELL* gene family. The HD consists of three helices and is conserved in eukaryotes and is involved in DNA binding [9,68]. Members in the same subfamily contain similar numbers of exons and introns, except for the number of exons in *P. patens*, which may also be due to the absence of exons for functional adaptation during evolution. Intriguingly, most members of the *KNOX* gene family contained five identical conserved motifs, except that *P. patens* does not contain motif 8. A high degree of sequence identity and similar exon–intron structures of *KNOX* genes across families suggests that the *KNOX* family has undergone gene duplication events throughout evolution.

Although repeated genes may have evolved few novel functions, they play an important role in the origin of species and the evolution of biological functions [42,69]. Gene duplication plays a significant role not only in the process of genome rearrangement and expansion but also in the diversification of gene functions and the large number of gene families [70]. Segmental duplication, tandem duplication, and transposition events, such as retro and replicative transposition, are the three main forces that drive the expansion of gene families [61,70]. Transposition events are difficult to identify based on sequence analysis alone; therefore, we focused on segmental and tandem duplication events. The results of the present study indicated that tandem duplication was detected in three species from the class I subfamily, indicating that the genes produced by tandem duplication did not undergo functional divergence (Table A3). Segmental duplication was detected in eight species from both subfamilies. Monocotyledonous plants had both tandem and segmental duplication, and dicotyledonous plants had only segmental duplication, especially, most of the *KNOX* genes in soybean and cotton. Therefore, the main amplification method of soybean and cotton is segmental duplication. Segmental duplication is likely to have played a pivotal role in *KNOX* gene expansion in dicots. In addition, the number of segmental duplication events in the two subfamilies was similar, and only segmental duplication occurred in the class II subfamily, which may be the reason for the larger number of class I subfamily members. Class I subfamily members are isolated from different angiosperms. They are expressed in meristems and not in differentiated tissues or organs that are related to maintaining the properties of meristems [9]. Large-scale duplication may have also been involved in the expansion of the *KNOX* gene family.

### 4.2 Functional divergence, positive selection, and co-evolution analysis

We selected the 3D structure of the *Arabidopsis KNOX* protein AT4G08150 for observation and marked the detected sites on the predicted 3D structure. The functional diversity between different subfamilies was mainly determined by specific amino acids in the subfamily, and the major reason for the functional divergence in repeated genes was possibly owing to the accumulation of amino acid mutation sites [42,71,72]. Type I and type II functional divergences between gene clusters of *KNOX* subfamilies were estimated by posterior probability analysis. By analyzing the functional divergence of the *KNOX* gene family, we identified a total of 15 type I functional divergence sites and 52 type II functional divergence sites from two subfamilies (Table 2). This result indicates diversification in the evolutionary rates of specific amino acid sites or significant changes in the physicochemical properties of amino acids. There are far more type II than type I functional divergence sites, which indicated that type II functional divergence is dominant. Besides, eight amino acid sites were identified as both type I and type II functional divergence sites, indicating that they had undergone simultaneous shifts in evolutionary rates and physicochemical properties. However, the lack of significant differences in the degree of functional divergence between most subfamily pairs suggests that genes belonging to these subfamilies might perform similar functions.

Positive selection has been associated with gene duplication and functional divergence. Here, we used computer simulations to evaluate the performance of Bayesian predictions for amino acids under positive selection [50]. However, the functions of most amino acid residues were conserved, and only a few amino acid sites can function in molecular adaptation [73]. In the present study, we detected 14 positive selection sites, three significant positive selection sites, and 11 extremely significant selection sites (Table 3). The positive selection sites were mainly distributed in the *KNOX1* domain, indicating that the *KNOX* gene family had been subjected to different selection pressures during evolution. Interestingly, we found that the site 140A experienced both type I and type II functional divergences and positive selection and may have an important role.

The complexity of protein evolution is directly proportional to the potential function and structure of interactions between co-evolving sites within the molecule, and co-evolving amino acid sites interact between complex functional domains of a protein [74]. Detection of co-evolving sites will provide important evidence for the study of the mechanisms underlying molecular evolution. A co-evolution analysis of the *KNOX* gene family detected two sets of adjacent co-evolving sites: 248S and 249D, and 382L and 383Y, both of which were in the c-terminal domain. Based on the analysis of the 3D structure (Figure 3), the co-evolving sites are closer in the 3D structure. Thus, the interaction of these sites may have a stabilizing effect on the spatial structure of *KNOX* proteins.

### 4.3 Expression analysis of *KNOX* gene family

Most *KNOX* gene families exhibited variable expression levels in different tissues and organs (Figures 4 and 5). Class I *KNOX* genes are mainly expressed in the SAM and the class II genes are more widely expressed [9], as can be seen in the heatmap, which shows members of the class II subfamily expressed in most tissues and organs. The expression of *KNOX* genes mainly in the shoot may be related to important role within the SAM [29]. For example, the expression of *KNOX* genes in the shoot was upregulated in *Arabidopsis*. In addition, during the evolution of angiosperms, the *KNOX1* gene was involved in the control of leaf shape. The expression pattern of *KNOX1* in the primordium of a leaf is highly related to the shape of the leaf. We suspect that the key amino acid sites in the *KNOX1* domain may be related to the expression of *KNOX1* [16]. The expression of genes in different tissues reflects the diversity of functions. In summary, expression profiles of *KNOX* family members are largely organ specific, indicating that *KNOX* genes are differentially expressed in different groups and that regulatory regions of *KNOX* genes may have diverged. Importantly, the results also demonstrate divergence in the expression of *KNOX* duplicated genes during evolution.

## 5 Conclusions

In the present study, a total of 129 *KNOX* family members were identified from 10 species through extensive analysis of gene families, which were divided into two subfamilies by phylogenetic analysis. Monocots and dicots were amplified differently. Both tandem and segmental duplication are found in monocotyledonous plants, whereas dicotyledonous plants only have segmental duplication. Gene replication provides the main driving force for adaptive evolution of species. The large proportion of type II functional divergence that occurred indicated that the mode of functional divergence for *KNOX* proteins mainly relates to changes in the physicochemical properties of amino acids. The site-specific model analysis revealed that the *KNOX* gene family contains 14 positive selection sites, mainly located in the *KNOX1* domain, which suffers from strong positive selection pressure. Two pairs of amino acid sites close to each other in 3D structure were identified by co-evolutionary analysis, indicating that they may play a key role in the stability of *KNOX* protein structure and function. Furthermore, *KNOX* genes exhibited different expression profiles in different organs as well as different functions. Our study provides a deeper understanding of the structural and functional evolution of the *KNOX* gene family and provides a basis for further research on *KNOX* proteins.

## Appendix

**Table A1:** Number of the *KNOX* genes in ten plant species

Plant taxa	Species	Number of *KNOX* genes
Dicotyledonous angiosperms	*Arabidopsis thaliana*	8
	*Glycine max*	29
	*Populus trichocarpa*	15
	*Gossypium raimondii*	21
	*Solanum lycopersicum*	8

Monocotyledonous angiosperms	*Oryza sativa*	11
	*Brachypodium distachyon*	11
	*Sorghum bicolor*	9
	*Zea mays*	13

Bryophyte	*Physcomitrella patens*	4

**Table A2:** Identified *KNOX* genes and their related information

Group	Gene ID	ORF (aa)	pI^a^	Mw (Da)
I	AT4G08150	398	6.02	45,835.5
I	AT1G70510	310	4.9	35,638
II	AT5G25220	431	5.86	47,599.8
II	AT5G11060	393	5.87	44,385
II	AT4G32040	383	6.03	43,283.6
I	AT1G23380	329	4.92	37,189.9
I	AT1G62360	382	6.19	42,753.1
II	AT1G62990	291	6.1	32,908.1
I	Glyma14g10430	385	6.39	44,100
I	Glyma0041s00360	387	6.43	44,458.4
I	Glyma04g05210	361	6.55	42,059.2
I	Glyma17g01370	269	6.46	30,750.9
I	Glyma09g01000	379	6.07	42,374.1
I	Glyma15g11850	375	5.83	41,992.8
I	Glyma07g39350	362	6.18	40,816.2
I	Glyma01g03450	309	5.22	35,125.5
I	Glyma02g04190	308	5.37	34,903.3
I	Glyma08g39170	321	4.99	36,284.8
I	Glyma18g20293	324	5.25	36,864.4
I	Glyma14g05150	311	6.72	35,759.5
I	Glyma20g22986	307	4.97	34,913.5
I	Glyma10g28820	296	5.06	33,654
I	Glyma19g41610	311	5.62	35,503.1
I	Glyma03g39041	307	5.6	35,332
I	Glyma04g35850	278	5.96	31,949.2
II	Glyma13g22530	346	5.59	39,797.2
II	Glyma17g11330	345	5.59	39,683.1
I	Glyma02g43761	295	5.7	33,383.3
II	Glyma04g06810	411	5.73	45,982
II	Glyma09g12820	369	5.51	41,611.2
II	Glyma06g06886	398	5.9	44,607.6
II	Glyma01g42410	281	6.43	32,145.4
II	Glyma11g02960	279	6.36	31,898.1
II	Glyma14g13750	412	5.67	45,926.9
II	Glyma05g03650	293	6.34	33,241.4
II	Glyma17g14180	292	6.24	33,080.3
II	Glyma17g32980	411	5.94	46,083.5
I	Potri.002G113300	368	5.98	42,459.4
I	Potri.011G011100	373	6.13	41,549.5
I	Potri.004G004700	369	6.14	41,189.2
I	Potri.015G079100	347	5.03	38,787
I	Potri.010G043500	309	4.88	35,200.6
I	Potri.012G087100	340	4.95	37,951.3
I	Potri.008G188700	341	5.51	38,685.4
I	Potri.005G017200	316	5.31	36,277.5
I	Potri.005G014200	317	5.31	36,324.5
I	Potri.013G008600	320	5.15	36,167.5
II	Potri.006G259400	432	5.91	48,185.5
II	Potri.018G114100	334	5.74	38,045.4
II	Potri.018G022700	426	5.88	47,762.8
II	Potri.001G112200	301	6.23	33,920.2
II	Potri.006G190000	338	5.81	38,563.1
I	Gorai.009G223200	369	5.8	42,516.5
I	Gorai.010G029000	364	6.14	41,729.5
I	Gorai.005G098100	310	7.1	35,620.9
I	Gorai.011G011700	351	6.01	39,746.5
I	Gorai.010G183800	355	6.21	39,951.8
I	Gorai.005G180500	314	5.01	35,440.9
I	Gorai.009G181500	353	6	39,924.9
I	Gorai.009G336900	313	4.73	35,622.3
I	Gorai.013G129400	312	5.17	35,341.7
I	Gorai.008G296800	303	5.47	34,206.5
I	Gorai.007G306500	320	4.73	36,277.4
I	Gorai.004G236400	289	5.66	33,046
II	Gorai.009G060200	425	5.79	46,817.2
II	Gorai.010G117100	434	5.89	48,085.5
II	Gorai.008G242800	303	6.5	34,634.2
II	Gorai.001G035700	443	5.94	48,698
II	Gorai.003G163800	299	5.9	33,596.7
II	Gorai.010G056100	290	6.2	32,792.9
II	Gorai.004G206600	300	6.23	33,765
II	Gorai.013G213900	448	6.01	49,635.2
II	Gorai.009G012300	295	5.35	33,294.4
I	Solyc04g077210.2	355	5.86	40,093.7
I	Solyc02g081120.2	354	5.72	39,654.6
I	Solyc05g005090.2	320	4.79	36,695.8
I	Solyc01g100510.2	335	5.3	37,912.2
II	Solyc08g041820.2	349	5.37	39,649.4
II	Solyc08g080120.2	310	6.06	35,307.6
II	Solyc07g007120.2	431	5.67	48,015.7
II	Solyc12g010410.1	329	5.58	38,098.1
I	LOC_Os03g51690	361	6.37	39,898
I	LOC_Os07g03770	355	6.5	38,590.4
I	LOC_Os03g56110	341	6.31	37,235.7
I	LOC_Os01g19694	301	6.13	32,735.8
I	LOC_Os05g03884	311	5.21	33,348.2
I	LOC_Os03g51710	377	5.67	41,382.8
I	LOC_Os03g56140	385	6.41	41,456.7
II	LOC_Os08g19650	278	5.8	30,891.6
II	LOC_Os02g08544	313	5.72	33,875.9
II	LOC_Os06g43860	323	6.02	35,106.2
II	LOC_Os03g03164	314	5.73	34,607
I	Bradi1g10047	372	6.38	41,486.1
I	Bradi1g57607	321	6.2	35,556
I	Bradi1g07247	345	6.13	38,136.9
I	Bradi2g11540	290	5.6	32,008
I	Bradi2g38390	300	5.61	32,991.1
I	Bradi1g12690	313	5.49	34,969.6
I	Bradi1g12677	251	6.61	28,353.8
II	Bradi3g19927	368	5.49	39,693.3
II	Bradi1g30730	317	5.69	34,713.7
II	Bradi3g06170	316	5.72	34,253.3
II	Bradi1g76970	314	5.82	34,834.4
I	Sb02g002200	356	6.23	38,884.6
I	Sb01g009480	360	6.56	39,863.8
I	Sb01g006790	349	5.96	38,713.5
I	Sb01g009460	372	6.29	40,540.6
I	Sb03g012480	294	5.6	32,458.4
I	Sb01g012200	334	5.53	36,797.8
I	Sb09g002520	303	5.34	32,961.1
II	Sb10g025440	319	5.79	34,605.6
II	Sb04g005620	444	6.34	48,081.1
I	GRMZM2G028041	351	6.4	38,800.6
I	GRMZM2G452178	360	6.23	39,215
I	GRMZM2G017087	359	6.41	39,826.6
I	GRMZM2G135447	364	5.91	40,276.2
I	GRMZM2G061101	352	6.53	38,968.6
I	GRMZM2G002225	328	5.25	36,438.7
I	GRMZM2G094241	307	5.27	33,326.4
I	GRMZM2G087741	295	5.51	32,493.4
I	GRMZM5G832409	298	5.32	32,662.6
II	GRMZM2G055243	316	5.69	34,522.6
II	GRMZM2G370332	310	5.84	33,791.9
II	GRMZM2G433591	363	9.78	39,958.3
II	GRMZM2G159431	310	5.73	34,336.7
I	Pp1s235_27V6	508	5.55	57,539.1
I	Pp1s33_357V6	445	5.44	50,715.3
II	Pp1s154_83V6	636	5.59	72,405.5
II	Pp1s77_59V6	518	5.24	58,858.8

ORF = open reading frame; aa = amino acids; Mw = molecular weight.

a Isoelectric point of the deduced polypeptide.

**Table A3:** Estimates of the dates for the segmental duplication events of *KNOX* gene family in eight plants

Segmental pairs		*K*_s_ (mean ± SD)	Estimated time (MYA)	WGD (MYA)
AT5G11060	AT5G25220	0.765 ± 0.129	25.5	28–48
Glyma04g05210	Glyma0041s00360	0.664 ± 0.171	54.4	13, 59
Glyma14g10430	Glyma0041s00360	0.125 ± 0.039	10.2	
Glyma01g03450	Glyma02g04190	0.181 ± 0.165	14.8	
Glyma01g03450	Glyma08g39170	0.696 ± 0.169	57	
Glyma01g42410	Glyma05g03650	0.709 ± 0.111	58.1	
Glyma01g42410	Glyma11g02960	0.147 ± 0.142	12	
Glyma01g42410	Glyma17g14180	0.711 ± 0.127	58.3	
Glyma02g04190	Glyma08g39170	0.664 ± 0.146	54.4	
Glyma02g04190	Glyma18g20293	0.605 ± 0.035	49.6	
Glyma02g43761	Glyma14g05150	0.237 ± 0.220	19.4	
Glyma03g39041	Glyma10g28820	0.606 ± 0.136	49.7	
Glyma03g39041	Glyma19g41610	0.144 ± 0.071	11.8	
Glyma03g39041	Glyma20g22986	0.610 ± 0.154	50	
Glyma04g05210	Glyma14g10430	0.613 ± 0.170	50	
Glyma04g06810	Glyma06g06886	0.159 ± 0.119	13	
Glyma04g06810	Glyma17g32980	0.633 ± 0.045	51.9	
Glyma05g03650	Glyma11g02960	0.766 ± 0.103	62.8	
Glyma05g03650	Glyma17g14180	0.163 ± 0.059	13.4	
Glyma06g06886	Glyma17g32980	0.620 ± 0.029	50.8	
Glyma07g39350	Glyma09g01000	0.646 ± 0.142	53	
Glyma07g39350	Glyma15g11850	0.705 ± 0.164	57.8	
Glyma07g39350	Glyma17g01370	0.145 ± 0.120	11.9	
Glyma09g01000	Glyma15g11850	0.183 ± 0.157	15	
Glyma09g01000	Glyma17g01370	0.659 ± 0.168	54	
Glyma10g28820	Glyma19g41610	0.572 ± 0.082	46.9	
Glyma10g28820	Glyma20g22986	0.145 ± 0.059	11.9	
Glyma11g02960	Glyma17g14180	0.741 ± 0.113	60.7	
Glyma13g22530	Glyma17g11330	0.153 ± 0.106	12.5	
Glyma14g13750	Glyma17g32980	0.158 ± 0.045	13	
Glyma15g11850	Glyma17g01370	0.660 ± 0.178	54.1	
Glyma19g41610	Glyma20g22986	0.573 ± 0.101	47	

Potri.005G014200	Potri.013G008600	0.313 ± 0.113	17.2	8–13
Potri.005G017200	Potri.013G008600	0.332 ± 0.111	18.2	
Potri.006G190000	Potri.018G114100	0.241 ± 0.065	13.2	
Potri.006G259400	Potri.018G022700	0.294 ± 0.115	16.2	
Potri.008G188700	Potri.010G043500	0.268 ± 0.094	14.7	

Gorai.001G035700	Gorai.010G117100	0.515 ± 0.130	17.2	13–20
Gorai.001G035700	Gorai.013G213900	0.530 ± 0.100	17.7	
Gorai.001G035700	Gorai.009G060200	0.495 ± 0.105	16.5	
Gorai.003G163800	Gorai.004G206600	0.535 ± 0.122	17.8	
Gorai.003G163800	Gorai.008G242800	0.626 ± 0.153	20.9	
Gorai.004G206600	Gorai.008G242800	0.534 ± 0.109	17.8	
Gorai.004G236400	Gorai.013G129400	0.420 ± 0.078	14	
Gorai.004G236400	Gorai.007G306500	0.519 ± 0.135	17.3	
Gorai.005G098100	Gorai.010G029000	0.710 ± 0.127	23.7	
Gorai.005G098100	Gorai.009G223200	0.528 ± 0.078	17.6	
Gorai.007G306500	Gorai.013G129400	0.425 ± 0.045	14.2	
Gorai.009G012300	Gorai.010G056100	0.484 ± 0.124	16.1	
Gorai.009G060200	Gorai.010G117100	0.530 ± 0.088	17.7	
Gorai.009G060200	Gorai.013G213900	0.526 ± 0.112	17.5	
Gorai.009G181500	Gorai.010G183800	0.482 ± 0.132	16.1	
Gorai.009G181500	Gorai.011G011700	0.617 ± 0.171	20.6	
Gorai.009G223200	Gorai.010G029000	0.637 ± 0.152	21.2	
Gorai.010G183800	Gorai.011G011700	0.683 ± 0.233	22.8	

LOC_Os02g08544	LOC_Os06g43860	0.660 ± 0.128	50.8	30–40,66–70
Bradi1g30730	Bradi3g06170	0.863 ± 0.017	66.4	56–73
Sb04g005620	Sb10g025440	0.685 ± 0.140	52.7–56.1	70
GRMZM2G135447	GRMZM2G452178	0.597 ± 0.024	45.9	12, 70

MYA = million years ago.
